# A Device for Characterizing Skin Physiological Response to Mechanical Loading in Transtibial Prosthesis Users

**DOI:** 10.3390/s26082288

**Published:** 2026-04-08

**Authors:** Molly E. Baumann, Mathew J. Weissinger, Joseph L. Garbini, Conor L. Lanahan, Joseph C. Mertens, Bailey Ramesh, W. Lee Childers, Joan E. Sanders

**Affiliations:** 1 Extremity Trauma and Amputation Center of Excellence, Defense Health Agency, Falls Church, VA 22042, USA; molly.baumann.civ@health.mil (M.E.B.); walter.l.childers.civ@health.mil (W.L.C.); 2 Department of Rehabilitation Medicine, Center for the Intrepid, 3551 Roger Brooke Drive, JBSA Fort Sam Houston, San Antonio, TX 78234, USA; 3 Department of Bioengineering, University of Washington, 355061, 3720 15th Ave NE, Seattle, WA 98195, USA; mweissin@uw.edu (M.J.W.); clanahan@uw.edu (C.L.L.); joemert@uw.edu (J.C.M.); bramesh@uw.edu (B.R.); 4 Department of Mechanical Engineering, University of Washington, 352600, 3720 15th Ave NE, Seattle, WA 98195, USA; garbini@uw.edu

**Keywords:** skin adaptation, amputee, interface stress, collagen, vasculature, optical coherence tomography, infrared imaging, thermal recovery, angiography

## Abstract

The skin’s physiological response to repetitive stress is not well understood in prosthesis users. Improving this understanding could facilitate the design of a diagnostic tool to determine if the skin is adapting to tolerate stress from a prosthetic socket. The objective of this research was to develop a physical system that mechanically stresses the skin in a controlled manner and then implements the imaging modalities of infrared (IR) imaging and optical coherence tomography (OCT). IR imaging characterizes the skin’s temperature response, while OCT characterizes vessel diameter changes over time in the skin. The system was implemented in a single individual with a transtibial amputation. The system reliably maintained the force profile throughout testing. IR and OCT imaging were initiated after load application, and all curves demonstrated an initial rise in temperature immediately after load removal followed by a decrease towards baseline. The system was able to effectively detect a peak outcome (temperature and vessel area) with both imaging modalities. The system’s ability to maintain the loading throughout and begin imaging to capture the peak provides promise for expanded use to better understand the skin’s physiological response to loading in prosthesis users. This improved understanding may better inform treatment strategies to optimize patient outcomes.

## 1. Introduction

For people with limb amputation to use a prosthesis, the skin on their residual limb must adapt to tolerate the continual mechanical stresses applied by the prosthetic socket. Pressures on the residual limb have been found to range from 12.5 kPa to 760 kPa [1], far greater than the stresses normally experienced on the lower leg during daily activity in able-bodied individuals. For the over 18,000 Military Health System beneficiaries who acquired a transtibial amputation from 2005 to 2023 [2] and the ~190,000 civilians who acquire a lower leg amputation annually [3], the failure of the residual limb skin to adapt and tolerate these loads can cause skin injury and limit prosthesis use until the damaged tissue heals. Limitations to prosthesis use can prohibit activity participation and impact overall quality of life.

Skin adaptation is a complex phenomenon that is not well defined. While there is ample evidence that skin reorganizes its collagen micro-architecture according to the mechanical stress to which it is subjected [4,5,6], vascular adaptations are less characterized. Vascular adaptations are important, as blood oxygenates the tissue and delivers the proper nutrients while removing metabolic wastes, a requirement for the tissues to survive. During mechanical stress, blood flow can be restricted, leading to a build-up of lactic acid and a loss of cardiac contractility [7]. Pressures as low as 4.3 kPa (32 mm Hg) can restrict blood flow and lead to pressure injury of the skin and surrounding tissue [8]. Mechanical stretching impacts the endothelial cells of the vasculature, as well as the extracellular matrix (ECM) supporting the vasculature. This leads to a reforming of the ECM and eventual angiogenesis to adapt to the mechanical stretch [9]. In physiological circumstances, this mechanical stretch is well handled by cell cascade pathways and helps to maintain proper circulatory function; however, if the mechanical stretch becomes pathological, it can lead to cell maladaptation and chronic conditions [9]. After brief ischemia, the circulation will attempt to re-perfuse the area with a variety of mechanisms proposed for instigating vasodilation and increased flow rate to return blood flow to the area rapidly—the post-occlusive reactive hyperemia response [10,11]. During this vasodilation, the vessel area will increase to allow for additional blood flow, which will impact skin temperature. The response to this ischemia is being more profoundly studied and used diagnostically to detect vascular issues sooner in pathophysiology [10], and has been noted as a potential indicator of overall limb health [12].

Adaptation of the reactive hyperemia response to the additional mechanical loads posed by prothesis use is expected to be essential in preventing skin breakdown. There is a balance between skin breakdown and adaptive changes to tolerate the higher loads that occur in response to mechanical stress [13]. In the short term, it is desirable to encourage adaptation, which requires mechanical stress to be applied, and finding the balance between breakdown and adaptation is vital. If the skin adapts to experiencing load, it can reduce injury, which would allow for continued prosthesis use. Quantifying this skin adaptation in prosthesis users may provide insight into better diagnosis and treatment strategies for improved outcomes. There is pilot data suggesting that the reactive hyperemia response occurs faster in prosthesis users [14]. The mechanical stress from the prosthetic socket should be inducing adaptation processes in the residual limb. Understanding differences in the response between a residual limb and the contralateral limb not subjected to these stresses could provide insight into the adaptation that is occurring and could allow for the diagnosis of residual limbs that are not adapting and are thus at a higher risk of skin injury. To achieve the ability to quantify the adaptation of the reactive hyperemia response, a system that delivers mechanical stress to the skin in a repeatable and controlled manner is necessary [15]. Therefore, the purpose of this research was to create a platform for characterizing residual limb skin physiological response to mechanical stress. The platform is a physical system that mechanically stresses the skin in a controlled manner and then quickly implements imaging modalities (infrared (IR) and optical coherence tomography (OCT)) that have shown promise towards the characterization of skin adaptation in rehabilitation applications [14,16,17,18,19,20]. Such a system could be used prior to prosthesis use to determine baseline response and to determine how best to tailor rehabilitation in socket wear to allow the skin to adapt. Information gained from utilizing such a platform would provide a better understanding of how the skin responds to mechanical stress and indicators of when skin is adapting or not adapting to the socket. In this paper, we describe the platform system, characterize its mechanical performance, and present post-load IR and OCT imaging results from the residual limb and contralateral limb of a single participant with a unilateral transtibial amputation to demonstrate a proof of concept for the further evaluation of skin response.

## 2. Materials and Methods

### 2.1. Load Applicator

A load applicator system was designed to position a 16 mm diameter loading pad on a region of skin on the lower limb of a human subject participant and apply an operator-selected cyclic pressure for an operator-specified time. Because, in our prior studies, we demonstrated collagen microarchitectural changes in skin using a comparable size loading pad [21,22], the relatively small surface area was considered acceptable for the purposes of this technology. The loading pad was machined from phenolic material to minimize any heat transfer from the load applicator to the skin. The system was capable of an upper limit of 500 kPa pressure, chosen based on measurements of limb–socket interface stresses reported in the prosthetics literature [1]. The load application assembly was designed so that it could be quickly moved out of the way and a thermal or optical camera, mounted to the support frame before loading is initiated, could be used to image the skin. The capability for imaging immediately after load application terminated was necessary because the early skin vascular response to mechanical stress was of primary interest. Thermal and optical imaging studies on the residual limbs of participants with limb amputation conducted by Swanson et al. [14] and Sanders et al. [23] demonstrated signal maxima less than a minute after load release at some locations. The inability to image quickly after load application limited data interpretation in a prior investigation [14].

#### 2.1.1. Hardware

**Figure 1:**
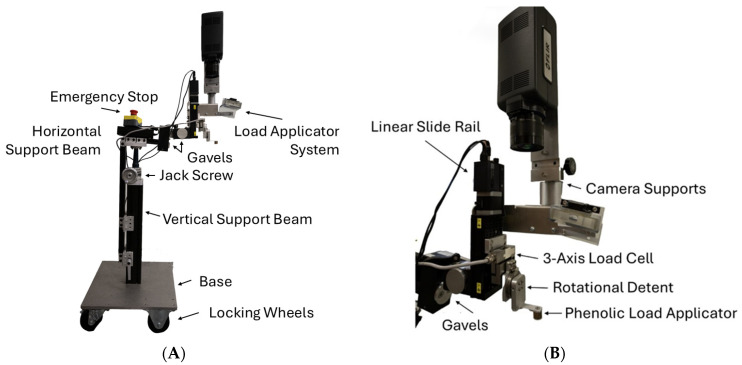
(**A**,**B**) Load applicator assembly. (**A**) The assembly is supported by an adjustable-height vertical support beam fastened to a heavy base with locking wheels. The gavels allow for adjustment of the pitch and yaw. (**B**) The load application system includes a phenolic load applicator, rotational detent, and 3-axis load cell. A linear slide rail moves the load application system up and down.

The load application system includes a linear slide rail (X-LSQ075B-E01, Zaber, Vancouver, BC, Canada), a 3-axis load cell (F3G-200N, Forsentek, Shenzhen, China), a rotational detent, and a phenolic applicator head, configured as illustrated in Figure 1A,B. The load applicator head material, which was phenolic, was specifically chosen to limit the thermal interference the test has on the physiological response observed. The system is compact so that the imaging system mounted to the camera support (IR or OCT) has a clear field of view as soon as the operator turns the rotational detent to move the loading head out of the way.

**Figure 2:**
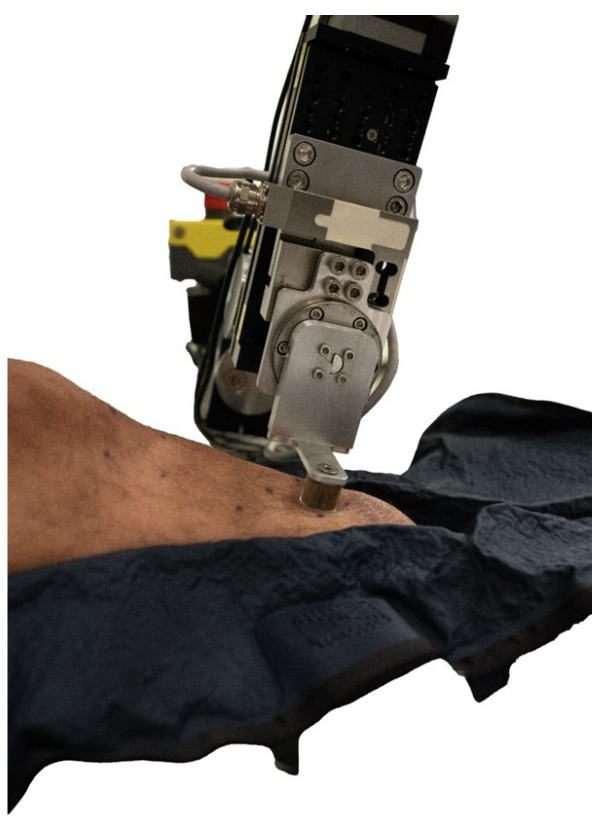
Load applicator aligned to participant’s residual limb for load application. The leg is vacuum-splinted to stabilize the limb and limit motion during loading.

The bottom of the base assembly is a 20 kg aluminum plate with four lockable wheels to stabilize the system during load application. A vertical support beam, locking jack screw, and horizontal support beam are fastened to the plate to allow the height of the horizontal support beam to be adjusted (~30 cm). The load application system is connected to the horizontal support beam through two gavels. The gavels allow for angular adjustment (60° of yaw, 20° pitch) so that the load applicator head can be positioned perpendicular to the participant’s limb axis and in the plane of the skin to ensure only perpendicular force, and minimal shear force is applied (Figure 2).

#### 2.1.2. Control System

The purpose of the load applicator controller is to produce a continuous sinusoidal variation in the vertical applied force with a specified amplitude and with a period of 1.0 s. In addition, the controller compensates for unwanted vertical motion of the skin surface by automatically adjusting the average position of the load applicator. Finally, if contact between the applicator and the skin is lost due to excessive skin motion, the controller automatically slews the applicator toward the skin at a constant speed until contact is restored.

A stepper motor motion controller moves the load applicator while a load cell continuously measures the applied vertical contact force. Before cyclic loading begins, displacement and force data are collected while the load applicator compresses the tissue at a constant rate. The skin surface stiffness is calculated by dividing the max force by the skin displacement, and the value is used in the execution of the dynamic control algorithm to maintain a consistent cyclic vertical force.

During active force control, the controller produces a sinusoidal motion profile with a specified amplitude. At the end of each 1 s motion cycle, the maximum and minimum force values during that cycle are assessed from the load cell measurements. These values are compared with the set point maximum and minimum forces, and a new motion profile that reduces the error is created for the next cycle. The effective settling time of the feedback controllers is approximately 6 s. Additional design information and a detailed engineering description of the control system are presented in Appendix A.

### 2.2. Imaging Modalities

The IR and OCT camera support assemblies were designed to allow up and down movement of the camera independent of the slide rail. The thermal camera support was designed to accommodate a larger adjustment range (~14 cm), while the OCT design prioritized finer adjustment due to the shorter focal length of the camera. An attachment for a smartphone IR imaging camera was also designed.

Two unique mounting brackets were designed to accommodate the different specifications and dimensions of the two different imaging modalities. The first was an IR mounting bracket that held both a stand-alone high-performance thermal camera and a smartphone thermal camera, while the second mounted an OCT camera.

The stand-alone high-performance thermal camera was a Science-Grade FLIR A6703 MWIR (Teledyne FLIR, Wilsonville, OR, USA). It operates in the 3.0–5.0 µm waveband and has a spatial resolution of 640 × 512 pixels, with each pixel being 15 µm. Still images or video files up to a 60 Hz sampling rate could be recorded. The temperature resolution was 0.018 °C. The attached lens had a focal length of 25 mm. In addition to the Science-Grade thermal camera, the custom mount included space for a Professional-Grade smartphone thermal camera (FLIR One Pro, Teledyne FLIR, Wilsonville, OR, USA). This camera had an accuracy of ±5% of the difference between the ambient and scene temperatures and a resolution of 160 × 120 pixels. Two thermal cameras were used to span the range of thermography technology.

The optical coherence tomography bracket allowed a Ganymede Series SD-OCT system (ThorLabs, Newton, NJ, USA) to be mounted. This system had an A-scan rate of 100 kHz, axial resolution of <3.0–6.0 mm, and central wavelength of 930 nm. An OCT-LSM03-BB lens was attached to the imaging unit. It had a lateral resolution of 8 mm, a focal length of 36 mm, and a field of view of 10 × 10 mm.

### 2.3. Standard Operating Procedure

At the start of a study visit, participants were asked to remove their prosthesis for at least 10 min to allow them to acclimate to the room environment. During this time, sites adjacent to the region of interest were marked on the residual limb and the contralateral limb using a surgical marker. The anatomical locations on the contralateral limb were matched to those of the residual limb. For participants with a transtibial amputation, sites included: the distal tibia proximal to the bone cut, the interosseous space between the tibia and fibula, and the mid-patellar tendon. Sites for participants with transfemoral amputation were: the lateral area of skin over the distal femur, the anterior midshaft of the femur on the amputated side, and the lateral area over the greater trochanter. Small fiduciary markers were made with thermal tape (3M High Contrast Thermal Tape 300LSE, IR.TOOLS, Crofton, MD, USA), creating a 2.5 × 2.5 cm square encompassing the area of load application contact with the skin. An identical square was marked directly adjacent, proximal and medial to the load-applicated region and was used as a reference area in the event that there were large temperature changes in the room during data collection that required thermal compensation strategies to be implemented during data processing.

The Science-Grade thermal camera was mounted 33 cm away from the surface of the skin. The lens was focused prior to image capture. To improve visualization, the thermal scale was locked prior to image capture to maintain the same color scale throughout each recording period. The low temperature was set to the temperature of the fiduciary markers. The high temperature was set 2.0 °C above the hottest observed region on the limb in the area of view prior to loading.

The participant laid supine in a comfortable position on a therapy table. Towels and foam blocks were used to support the participant’s legs. Once a comfortable position was achieved, vacuum splints were used to hold the leg stationary during load application and imaging to reduce motion artifacts in the data (Figure 2). The load applicator was positioned within the center of the region of interest, and alignment was verified through a custom-designed graphical user interface (GUI) that showed the applied three-dimensional force data on the screen. The load applicator was manually displaced downward by the operator to apply a 15 N force perpendicular to the skin surface (Z-direction). A 15 N force was used because, in pilot studies, this magnitude was shown to induce a strong skin vascular response. Adjustments were made to the gavels of the load applicator system to minimize loading in shear while maintaining the 15 N vertical force. Once the load applicator was positioned in the correct orientation, the load applicator was raised and brought to rest approximately 1 cm above the surface of the skin.

The load application process was started using a second custom-designed GUI. Before cyclic loading was started, to determine the skin tissue’s mechanical stiffness, the load applicator slowly ramped up to a 15 N applied force. It returned to its “home” position 2.0 mm above the surface of the skin, and the load applicator was rotated out of the way to clear the field of view for the camera so that pre-loading images of the skin could be recorded. Thermal images were recorded for 1 min (0.5 Hz). OCT images were collected as quickly as possible for 1 min, which normally generated two pre-loading images. After the pre-loading images were collected, the load applicator was rotated back into place, and load application commenced. A cyclic force from 5 N to 15 N (25 to 75 kPa) was applied in the Z-direction for 10 min (1 Hz). The Z-direction force profile was output to the screen to verify proper adjustment during the loading period. This force range was selected to induce a response similar to that seen in pilot testing. It is within the range that is reported for forces expected within the socket [1] and minimizes the risk of any lasting damage to the skin due to the inherent differences between individuals in response to load. Once completed, the load applicator was rotated out of the way again, and image acquisition was started. Images from the Science-Grade thermal camera were collected at 0.5 Hz for 10 min and stored in a single video file. The Professional-Grade thermal camera recorded continuous video for the 10 min period. OCT images were collected for 10 min, with images captured as quickly as the software would allow (typically 2 images/min). The focus of the OCT images was adjusted as needed throughout the image capture period.

### 2.4. Data Processing

Custom processing algorithms written in MATLAB (2022b, MathWorks, Natick, MA, USA) were used to reduce the data. Independent code was written to process the OCT, thermal, and load applicator data.

#### 2.4.1. Load Application Data

Load applicator position and force data were processed using a peak identification method. Maximum and minimum forces in all three axes, slide rail maximum and minimum positions relative to the displacement, and time between force maxima were identified throughout the loading protocol, and a mean and standard error relative to the set point were calculated for each of these variables.

#### 2.4.2. Thermal Image

The fiduciary markers were identified in the infrared thermal videos and used to register each individual frame, accounting for motion artifacts. Once registered, a circular region of interest (ROI) (40-pixel radius) was selected by the operator, and a mean was calculated from the pixels within the ROI. The mean temperature was plotted versus time (frame number) to create a temperature response curve. A sixth-order regression was fitted to the data so that the time to 70% peak temperature relative to the end of load application could be calculated.

#### 2.4.3. OCT Image

For OCT, the data reduction methodology from our prior work [19] was extended. High-sensitivity variance images were created from the five repetitive B-scans as described by Choi et al. [24] to create high-sensitivity OCT–angiography (OCTA) images. +Vessel area density (VAD) was calculated by thresholding the angiography image to a binary image and dividing the sum of the image by the total number of pixels. VAD was plotted for the time series of OCTA images to calculate a time-based response to the load application. Because of the low OCT sampling rate, the single highest VAD is reported rather than regression fitting the data.

### 2.5. Preliminary Study

The study protocol was approved by the San Antonio Institutional Review Board in compliance with applicable Federal regulations governing the protection of human subjects. The individual presented in this proof of concept had a unilateral transtibial amputation. The distal end of the tibia and the interosseous space between the tibia and fibula (IO) in the residual limb and the matched control sites in the contralateral limb were tested. The Standard Operating Procedure, as detailed above, for the load applicator was conducted, and IR and OCT imaging were conducted.

## 3. Results

During both perturbation experiments conducted on the intact and residual limbs, the load applicator system reliably maintained the maximal and minimal force set by the operator, 15.0 N and 5.0 N, respectively (Table 1).

**Figure 3:**
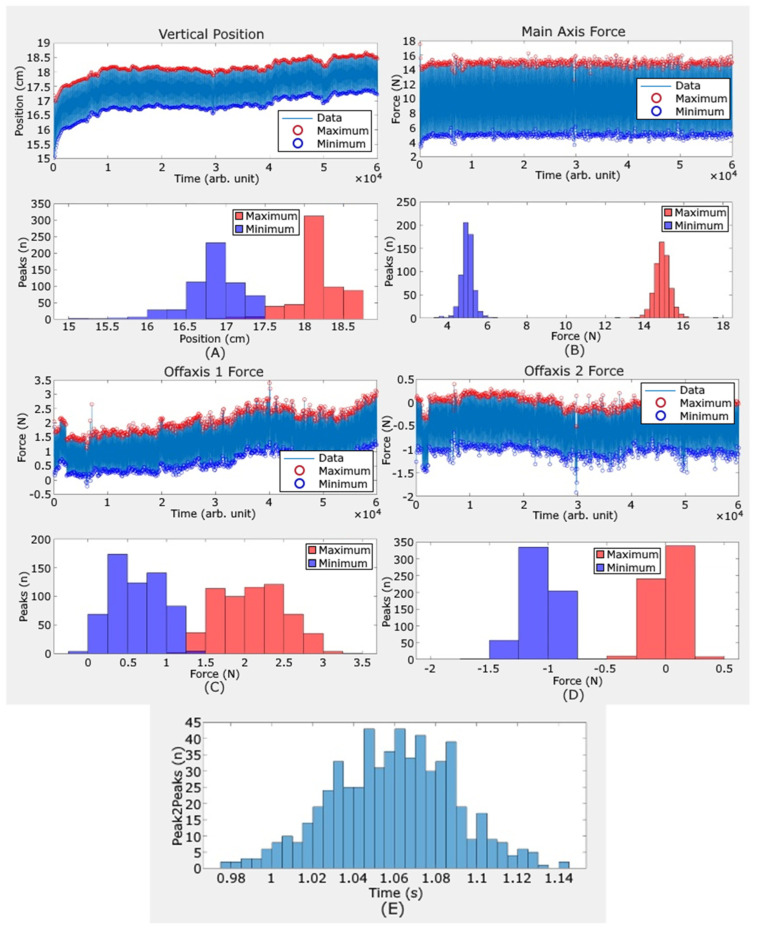
(**A**–**E**). Results from the interosseous space site of intact limb. (**A**) Over the 10 min loading period, the load applicator displaced upwards about 1.0 cm, possibly from participant movement during the test. (**B**) The maximum and minimum loads were well maintained near 15.0 and 5.0 N, respectively. (**C**,**D**) The off-axis loads were 2.2 N or less. (**E**) The time between peaks was well maintained near 1.0 s.

The IO site required greater load applicator head displacement to maintain the loading profile compared to the tibia. As shown in Figure 3A,B, the load applicator displaced upwards about 1.0 cm during the test to maintain the applied load. This is likely a response to the participant moving during the load application period. The mean off-axis loading was 2.2 N or less in both directions (Figure 3C,D). The time between peaks was well maintained (Figure 3E). Table 1 provides more detailed loading information.

The detent was rotated within 1–2 s after the test was completed, removing the load applicator head from the imaging field of view. The thermal camera was re-focused rapidly (within 2–3 s). The OCT requires a finer focus adjustment, which took more time to execute (20–30 s).

**Figure 4:**
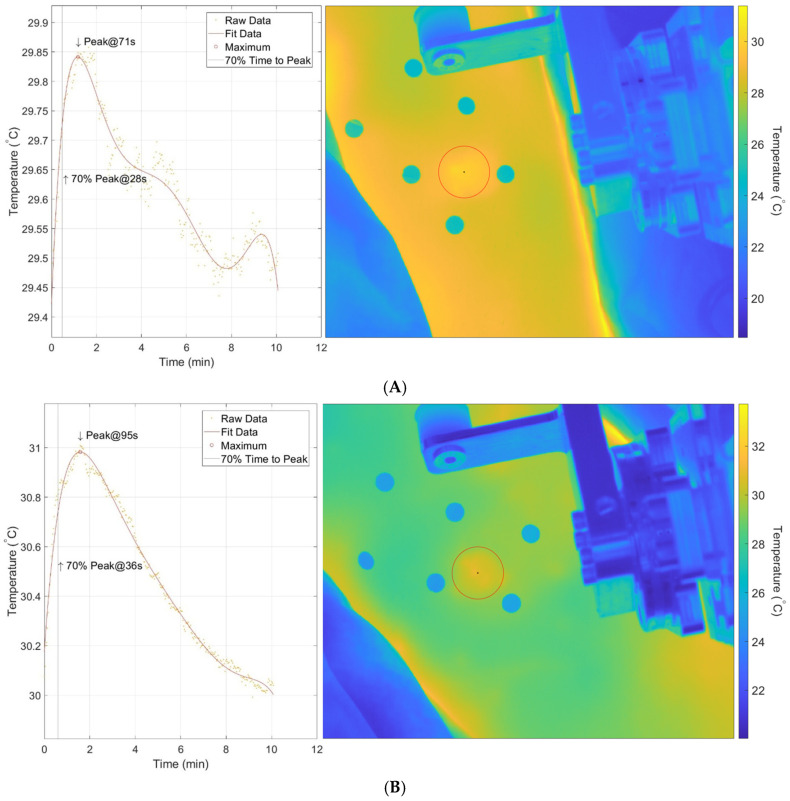
(**A**,**B**). Example results from the Science-Grade thermal camera. The data are fit with a 6th-order polynomial. Thermal response over time after loading and a thermal image (test site within red circle) are shown for (**A**) a tibia location and (**B**) an interosseous space location. Data are from the intact limb of participant. Arrows indicate calculated point of time to peak temperature and time to 70% of peak temperature.

The Science-Grade thermal camera video data was reduced using custom code. Figure 4A,B illustrate the thermal response versus time and the sixth-order regression fit to data from the tibia and IO sites, respectively. The maximal temperature response was identified, and the time to 70% peak was calculated. All curves demonstrated an initial rise in temperature after load removal, followed by a decrease in temperature toward its initial temperature after loading. The Science-Grade time to 70% peak ranged from 28 to 42 s across limbs (Table 2). The Professional-Grade thermal camera time to 70% peak ranged from 30 to 39 s across limbs (Table 2).

## 4. Discussion

The mechanical system designed in this study demonstrated the ability to reliably maintain the desired load for the duration of a testing session to stress the skin surface in a repeatable manner for the evaluation of skin adaptation via imaging using IR and OCT cameras. Skin adaptation to mechanical stress is an active area of research. Micro-architectural differences in skin between sites subjected to repetitive stress and those minimally stressed have been identified [4,6,25,26], suggesting that the skin will reorganize depending on its mechanical environment. There is a plethora of literature on the differences in structure between anatomical sites [13,27]. Collagen fibril diameters in various tissue were shown to increase and fibril volume density was shown to decrease in response to repetitive pressure and shear stress, suggesting architectural adaptation without an increase in collagen volume [21,28,29,30]. This collagen reorganization may be linked to other physiological changes such as vascular adaptations to mechanical stress. Pilot data demonstrating blood vessel area density increases in repetitively stressed skin have been reported [14,19], though methodological issues, primarily the inability to sample immediately after load release, limited the insight gained.

**Figure 5:**
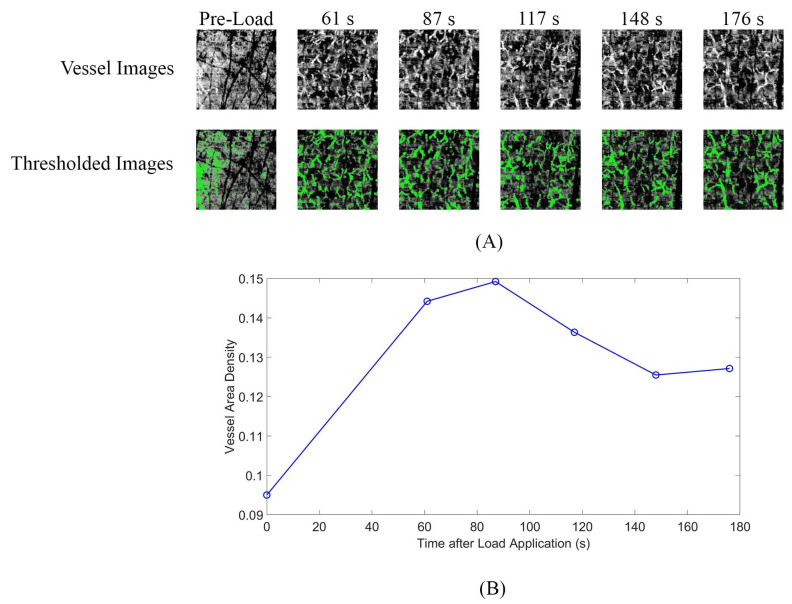
(**A**,**B**) Example results from optical coherence tomography (OCT) camera. (**A**) Vascular and thresholded images show identification of vessels in the pre-loading state and first five images post-loading. (**B**) Vessel area density (VAD) is plotted as the percent of the image identified as blood vessels. A small increase (0.5%) is identified at 87 s post-load, followed by a decrease (2.4%) at 148 s.

The OCT VAD curves showed a brief increase in VAD immediately after load application, followed by a decrease, similar to the thermal camera response curves (Figure 5A,B). The time to 100% peak for OCT imaging was lower than that for IR imaging at the IO location and greater than that for IR imaging at the tibia location (Table 2).

The developed system is an extension of a prior apparatus we developed to apply a consistent loading profile to the skin at anatomical points of interest despite participant movement shifts during testing [14]. The present system overcomes the previous challenges experienced attempting to image the skin immediately after the load is removed. The load applicator head was quickly moved out of view in the present study, 2–3 s for thermal imaging and 20–30 s for OCT, much faster than in our prior investigation, which used 54 s for OCT imaging [14]. We can now capture the maximum skin temperature and VAD after load release, which may be important for understanding post-occlusive reactive hyperemia [14,19]. In addition, the phenolic material in the present study limited the thermal interference from the load applicator head, improving the quality of the data. The mean coefficient of variability (standard deviation/mean) in the maximum force from cycle to cycle, 2.9%, and minimum force from cycle to cycle, 6.5%, is comparable to that in our prior system [22], indicating no loss in performance with the functional improvements. This highlights the capability of the system to reliably maintain the loading profile throughout testing.

While being from only a single participant, these results are promising for ongoing efforts utilizing this system for monitoring residual limb health. The response curves observed in this single-participant investigation show similarities to those reported in prior published research [14,19,31,32,33]. Given the similar curve profile results from the different imaging modalities, they are likely all measuring variables related to blood flow. Differences in the time to peak between the two IR cameras and the OCT may be explained by several factors: (1) The Professional-Grade camera has a poorer resolution than the Science-Grade thermal camera and may not be able to fully distinguish subtle changes in the temperature during recording. (2) The time to record and store a single OCT image is approximately 20 s (0.05 Hz sampling rate), which limits the ability to directly compare the time to peak of the thermal cameras, as those images are being collected at approximately 2 s intervals (0.5 Hz sampling rate). (3) IR imaging may be more strongly influenced than OCT by blood flow changes in deeper tissues.

This work demonstrates the possibility of monitoring residual limb health and allows for a comparison of responses between the residual and contralateral limb. Differences between the limbs can be monitored over time in the clinic, which allows for an improved detection and diagnosis of skin health issues that plague prosthesis users. Earlier detection may allow for preventative measures rather than treatments after the issue has progressed, requiring the individual to temporarily limit or stop altogether their prosthesis use while their limb heals. By maintaining healthy residual limbs, users can continue using their prosthesis, allowing for continued participation in activity and improving outcomes. This will require a clinically relevant measure that can be easily performed during a standard clinic visit. IR imaging is easier to implement in the clinic and requires less processing time than OCT, making it a potentially viable pathway to clinical monitoring. As the participant numbers increase in this study, differences in responses in the residual vs. contralateral limb may be better understood by continuing more extensive clinical implementation. Using the information gained through this study, ways to tailor the current protocol to be more easily implemented in the clinic can be evaluated. By performing these or similar measurements prior to socket use, the baseline health of the skin can be established, and rehabilitation treatment and socket wear can be tailored to avoid skin breakdown.

There are several limitations to this study. The current single-subject study has limited generalizability; however, the study is ongoing to recruit additional participants so that broader generalizations may be made. Additionally, as results from a larger population are captured, VAD and temperature results will be correlated to clinical outcomes to better understand how these measures relate to what is seen clinically. The OCT imaging delay may still cause some peak values to be missed. In this instance, discernable changes are seen in the recording time frame, but this is a limitation of the OCT system, as it takes time to focus, record, and store the image data. This study only evaluated axial forces. Future work should include a shear component, as it is considered of high clinical relevance in this population [34]. Shear stresses up to 57.0 kPa have been measured on prosthesis users [35]. Additional future work would expand the range of loading to encompass higher pressures. This work can serve as the baseline, and future studies can extend the maximum pressure to higher-activity pressures experienced in the prosthetic socket. This could involve a very similar methodology as presented here, but with a higher maximum pressure.

While this approach allows for rapid imaging after load application is complete, future work could consider strategies to image through the load applicator head to allow for imaging during the load application phase. The evaluation of blood return or pumping during the unweighting phase could further highlight changes in the physiological response of the residual limb to prosthesis wear. Previous studies have performed vasodilation analysis of OCT images, which can be done through a mineral oil container that is actively heating the skin [19]. This ability to image while loading opens avenues for future studies to better understand skin response during prosthesis wear. This framework could be used to monitor skin adaptation and residual limb health. These capabilities could be particularly useful to better evaluate how different treatment strategies impact skin health over time. Tracking skin health throughout treatment can better inform treatment strategies to optimize outcomes. Additionally, this system could help better establish baseline data of residual limb health for prosthesis users. Combined with current treatment interventions, prosthesis wear time, and other relevant factors, this information could help establish a database of residual limb health to inform clinical decision-making.

## Figures and Tables

**Table 1:** Load applicator position and force results from a participant’s residual limb and contralateral limb. Means and standard deviations from all session data are shown.

Imaging Modality	Anatomical Site	Pk-to-Pk Time (s)	Major Axis		Major Axis		Off-Axis 1		Off-Axis 2	
			Max Position (cm)	Min Position (cm)	Max Force (N)	Min Force (N)	Max Force (N)	Min Force (N)	Max Force (N)	Min Force (N)
Infrared (IR)	Interosseous Space Intact Limb	1.06 ± 0.018	18.2 ± 0.6	15.1 ± 0.7	14.9 ± 0.6	5.0 ± 0.3	−0.3 ± 0.2	−1.2 ± 0.4	1.1 ± 0.6	0.3 ± 0.4
	Interosseous Space Residual Limb	1.07 ± 0.028	23.2 ± 1.0	20.4 ± 1.1	14.9 ± 0.4	5.0 ± 0.3	2.2 ± 0.6	1.4 ± 0.6	−0.4 ± 0.3	−1.3 ± 0.5
	Tibia Intact Limb	1.06 ± 0.030	18.2 ± 0.3	16.8 ± 0.4	14.9 ± 0.4	5.0 ± 0.3	2.1 ± 0.4	0.6 ± 0.3	0.01 ± 0.1	−1.1 ± 0.1
	Tibia Residual Limb	1.06 ± 0.025	15.9 ± 0.4	14.0 ± 0.4	14.9 ± 0.3	5.0 ± 0.3	−0.2 ± 0.1	−0.8 ± 0.1	−0.2 ± 0.1	−1.4 ± 0.2
Optical Coherence Tomography (OCT)	Interosseous Space Intact Limb	1.07 ± 0.032	14.5 ± 0.2	12.2 ± 0.3	14.9 ± 0.4	5.0 ± 0.3	0.8 ± 0.2	0.2 ± 0.1	1.6 ± 0.5	0.9 ± 0.4
	Interosseous Space Residual Limb	1.07 ± 0.029	18.4 ± 0.6	15.3 ± 0.7	15.0 ± 0.4	5.0 ± 0.3	1.0 ± 0.3	0.3 ± 0.3	0.7 ± 0.4	−0.007 ± 0.5
	Tibia Intact Limb	1.07 ± 0.043	9.7 ± 0.5	8.0 ± 0.5	14.8 ± 0.7	4.9 ± 0.5	−0.5 ± 0.2	−1.7 ± 0.2	0.8 ± 0.3	0.2 ± 0.2
	Tibia Residual Limb	1.06 ± 0.029	12.6 ± 0.3	10.8 ± 0.3	14.9 ± 0.3	4.9 ± 0.3	0.7 ± 0.1	0.07 ± 0.06	1.2 ± 0.2	0.3 ± 0.1

**Table 2:** Time to peak temperature. Results from a participant’s residual limb and contralateral limb are shown.

Location	Infrared (IR)				Optical Coherence Tomography (OCT)
	70% Time to Peak (s)		100% Time to Peak (s)		100% Time to Peak (s)
	Science-Grade	Professional-Grade	Science-Grade	Professional-Grade	
Interosseous Space Intact Limb	36	32	95	84	76
Interosseous Space Residual Limb	42	39	101	89	69
Tibia Intact Limb	28	30	71	73	98
Tibia Residual Limb	38	34	103	89	151

**Figure A1:**
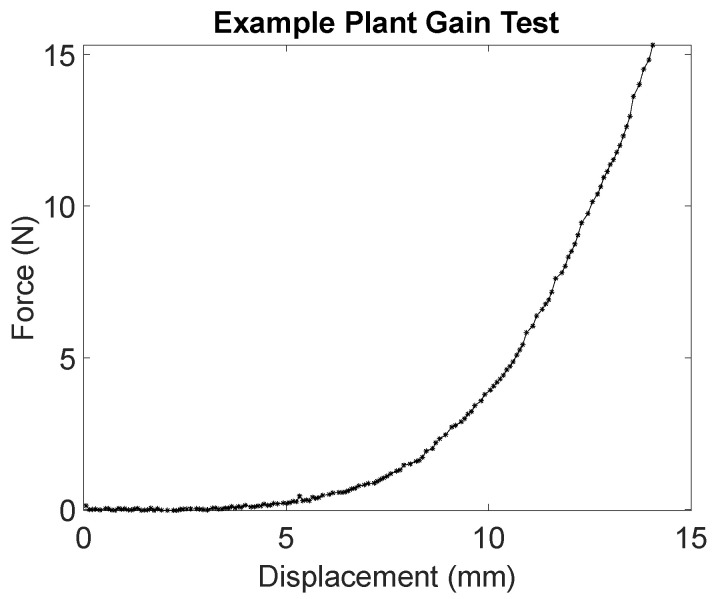
Example result from a plant gain test.

**Figure A2:**
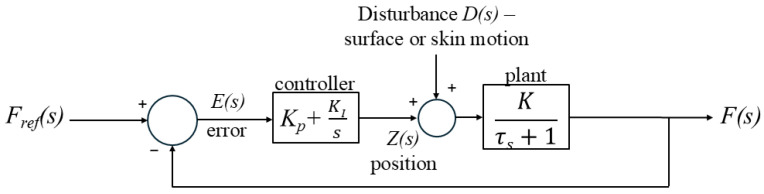
Control system block diagram.

## Data Availability

Data from this study are included in Supplementary Tables S1 and S2.

## Supplementary Materials

The following supporting information can be downloaded at: https://www.mdpi.com/article/10.3390/s26082288/s1, Table S1: Load applicator position and force results; Table S2: Time to peak temperature results.

## Abbreviations

The following abbreviations are used in this manuscript:

ECM	Extracellular matrix
GUI	Graphical user interface
IR	Infrared imaging
IO	Interosseous
OCT	Optical coherence tomography
OCTA	Optical coherence tomography–angiography
ROI	Region of interest
VAD	Vessel area density

## Appendix A

A three-axis load cell with a ±200 N range (F3G-200N, Forsentek, Shenzhen, China) was fixed to the linear stage using custom interfacing hardware to provide force feedback to the control system. The load cell signal was amplified to a range of ±10 V (FSC (−10 V–10 V) − 12 V, Forsentek, Shenzhen, China) and calibrated in all three axes using a 111.2 N weight (56% load cell capacity). Amplifier gains were adjusted to output 0.05 V/N, allowing for accurate force measures up to the full range of the load cell with no signal saturation. Force signal linearity was verified on each axis with a secondary 44.5 N weight (22.2% load cell capacity). The load cell output and amplifier voltage supply were read by the computer via a data acquisition device (USB-6002, National Instruments, Austin, TX, USA).

Because OCT imaging is highly sensitive to small motion artifacts, an imaging foot was used to apply a small configurable holding force, F_foot_, to the skin during OCT camera focusing and imaging as a means to improve the stability of the tissue. A detent mechanism was attached to the face of the load cell, which allowed for toggling between the loading head and the imaging foot. A small aperture in the imaging foot allowed the OCT camera to see the entire field of view while the surrounding tissue was held in place.

For thermal imaging collections, the imaging foot was unnecessary due to a lower focusing sensitivity and greater focal length. Additionally, the field of view of the thermal camera was much larger than that of the OCT camera. To avoid interference of the imaging foot in thermal images, it was removed from the assembly, and imaging foot holds were bypassed in the protocol for the thermal imaging mode.

The desired force output during cyclic load application was a 1 Hz sinusoidal force waveform with a trough value F_min_ and a peak value F_max_. To apply this cyclic load, the slide rail was commanded to perform a 1Hz sinusoidal position waveform with a manipulated trough value, z_min_, and manipulated peak value, z_max_. Sinusoidal positioning commands could easily be streamed to the slide rail with a configured amplitude and period using Zaber’s LabVIEW driver library. During each cycle, the slide rail was commanded to move to lz_min_. The sinusoidal position command was then sent with a period of 1 s and an amplitude of z_max_–z_min_, such that the position peaked at z_max_. Force data were sampled during this sinusoidal command, and the two extrema were pulled to define control error variables, e_min_ and e_max_.

Separate control systems were designed for controlling F_min_ and F_max_. The plant was modeled as a first-order system with a plant gain of K_min_ for the F_min_ controller and K_max_ for the F_max_ controller, expressed as stiffnesses in units of N/mm.

Plant gains for each controller were measured during a plant identification step in which the loading head was slowly depressed into the skin surface until the desired maximum force F_max_ was reached (Figure A1).

Displacement values of the samples closest to F_min_ and F_max_ were pulled from the force–displacement curve and used to estimate the minimum and maximum slide displacement of the sinusoidal positioning waveform, termed z_min-est_ and z_max-est_, respectively. These values were later used to initialize their respective controllers.

To calculate plant gains, the slope of the force–displacement curve was fit locally near F_min_ and F_max_ using these estimated positions. A linear regression was performed on the five nearest data points surrounding z_min-est_, and the slope was used to define K_min_. To estimate K_max_, the slope was taken from a linear regression performed on the last five samples taken during the plant identification step. Generally, K_max_ was found to be significantly larger than K_min_ due to an increase in tissue stiffness with increasing depth of the loading head into the tissue, supporting the need for separate plant gain calculations.

The PI controller was tuned to provide a damped control response. The closed-loop transfer function was translated into a discrete transfer function using Tustin’s method. This discrete system was later implemented in the protocol application using an infinite impulse response (IIR) filter.
